# Induction of plasmid-mediated AmpC β-lactamase DHA-1 by piperacillin/tazobactam and other β-lactams in *Enterobacteriaceae*

**DOI:** 10.1371/journal.pone.0218589

**Published:** 2019-07-08

**Authors:** Kentaro Akata, Tetsuro Muratani, Kazuhiro Yatera, Keisuke Naito, Shingo Noguchi, Kei Yamasaki, Toshinori Kawanami, Takashi Kido, Hiroshi Mukae

**Affiliations:** 1 Department of Respiratory Medicine, University of Occupational and Environmental Health, Japan, Kitakyushu City, Fukuoka, Japan; 2 Department of Clinical Microbiology, Kyurin Medical Laboratory, Kitakyushu City, Fukuoka, Japan; 3 Second Department of Internal Medicine, Nagasaki University School of Medicine, Nagasaki City, Nagasaki, Japan; Universita degli Studi di Torino, ITALY

## Abstract

Chromosomal AmpC β-lactamase induction by several types of β-lactams has been reported, but not enough data are available on DHA-1 β-lactamase, a plasmid-mediated AmpC β-lactamase. Therefore, we evaluated the DHA-1 β-lactamase induction by various antibiotics including piperacillin/tazobactam (PIP/TZB) in this study. Six strains (*Enterobacter cloacae* 2 strains, *Citrobacter freundii* 1 strain, *Serratia marcescens* 2 strain, and *Morganella morganii* 1 strain) possessing chromosomal inducible AmpC β-lactamase were used as controls. Four strains (*Escherichia coli* 2 strains, *Klebsiella pneumoniae* 1 strain, and *C*. *koseri* 1 strain) possessing DHA-1 β-lactamase were used. The β-lactamase activities were determined by a spectrophotometer using nitrocefin. β-lactamase induction by PIP, PIP/TZB was not observed in any strains and β-lactamase induction by third- and fourth-generation cephems was not observed in most strains. The induction ratios of the chromosomal AmpC β-lactamase in the reference group by PIP/TZB were <1.51, and those of the DHA-1 β-lactamase were <1.36, except for *K*. *pneumoniae* Rkp2004 (2.22). The β-lactamase induction by first- and second-generation cephems, flomoxef, and carbapenem differed in each strain. Cefmetazole (CMZ) strongly induced β-lactamase. This study demonstrated that the induction of DHA-1 β-lactamase was similar to that of chromosomal AmpC using various *Enterobacteriaceae*, although the induction of β-lactamase in both groups by PIP/TZB was low. We also reported that the induction of PIP/TZB, a β-lactamase inhibitor combination antibiotic, against various AmpC-producing *Enterobacteriaceae*, including DHA-1 producers, was low.

## Introduction

β-lactamase is a major factor involved in drug-resistance, and most Gram-negative rods have some genes encoding Ambler class A or C β-lactamases in their chromosomes [1]. Gram-negative bacteria such as *Enterobacter* spp., *Citrobacter freundii*, *Serratia* spp., *Providencia* spp., *Morganella morganii*, *Hafnia alvei*, *Aeromonas* spp., and *Pseudomonas aeruginosa* possess AmpC type β-lactamase in class C. In contrast, *Klebsiella* spp., *Salmonella* spp., *Citrobacter* spp. except for *C*. *freundii*, *C*. *murliniae*, *C*. *youngae*, and *C*. *werkmanii* and *Proteus mirabilis* do not possess AmpC type β-lactamase. In the process of AmpC β-lactamase synthesis, the *AmpR* can act as either a transcriptional repressor or an activator, depending on concentrations of precursor muropeptides. The synthesis of large quantities of AmpC β-lactamase is induced due to high concentrations of precursor muropeptides in the presence of β-lactam antibiotics [2–5].

Some bacteria lacking genes that can induce β-lactamase in their chromosomes still manage to achieve the ability to produce β-lactamase through gene transmission via plasmid [6–8]. Six plasmid-type AmpC β-lactamases with differing genetic backgrounds have been reported, including ACT type from *Enterobacter* spp., CIT type from *C*. *freundii*, DHA type from *M*. *morganii*, ACC type from *H*. *alvei*, and MOX and FOX types from *Aeromonas* spp. [9]. Unlike bacteria with native genes encoding β-lactamase, with the exception of DHA, these bacteria lack or *ampR* (or a functional *ampR*); thus, the enzymes are constantly produced by these bacteria [10]. However, plasmids carrying bla DHA-1 also carry *ampR* and hence the β-lactamase is inducible [11]. Muratani et al. first reported DHA-1-producing *K*. *pneumoniae* in 2006 in Japan [12], and Yamasaki et al. reported that the prevalence of DHA-1-producing *K*. *pneumoniae* was 0.1% (6/5,970) in the Kinki region of Japan in 2010 [13]. The chromosomal AmpC β-lactamase-based induction of several types of β-lactams have been reported [14,15], but there are not enough data concerning the difference of the DHA-1 β-lactamase-based induction by each anitibiotics although there are only 2 reports (the reports of Barnaud et al. [11]. and Poirel et al. [16]). Barnaud et al. reported β-lactamase induction by imipenem in transconjugant *E*. *coli* HB101 containing pSAL2-ind [11]. Poirel et al. reported β-lactamase induction by various antibiotics in *E*. *coli* DH10B harboring recombinant plasmid pPON-1 [16]. Both reports only investigated transconjugant *E*. *coli*. However, we think that the difference in β-lactamase induction by various antibiotics in many clinical strains of *Enterobacteriaceae* is important.

Regardless of the presence of inducible AmpC β-lactamase on a chromosome, *Enterobacteriaceae* strains are able to acquire DHA-1 β-lactamase because most DHA-1 β-lactamase is encoded on a plasmid. When choosing antibiotics against infectious diseases caused by organisms such as *E*. *coli*, *K*. *pneumoniae*, and *P*. *mirabilis* that do not have inducible AmpC β-lactamase on their chromosomes, it is important to consider whether or not the organism has acquired DHA-1 β-lactamase. It is also important to understand the DHA-1 β-lactamase induction of various β-lactams, especially piperacillin/tazobactam (PIP/TZB).

PIP/TZB is one of most popular parenteral β-lactams for use against various bacterial infections caused by *Enterobacteriaceae* in Japan; however, there are not enough data concerning AmpC β-lactamase and DHA-1 β-lactamase induction. Therefore, we evaluated the induction of various β-lactams, including PIP/TZB, by several AmpC β-lactamases or DHA-1 β-lactamase.

## Materials and methods

### Bacterial strains

Six strains (the *E*. *cloacae* ENel67 and ENel223 strains, *C*. *freundii* Rcf53 strain, *Serratia marcescens* ENsm22 and ENsm202 strains, and *M*. *morganii* ENmm80148 strain) were used as controls or chromosomal AmpC producers in this study. Four strains (the *E*. *coli* Mec3968 and Mec5372 strains, *K*. *pneumoniae* Rkp2004 strain, and *C*. *koseri* Rck4438 strain) were used as DHA-1 β-lactamase producers. All bacterial strains are clinical strains. These 10 strains were selected from clinical isolates obtained from different patients between 1999 and 2010 from various hospitals on northern Kyushu island, Japan.

### Antibiotics

β-lactam antimicrobials, which is antimicrobials of defined potency, were obtained from their respective manufacturers as follows: 1) penicillin antibiotics—piperacillin (PIPC; Tokyo Kasei, Tokyo, Japan) and piperacillin/tazobactam (PIP/TZB; Toyama Chemical Co., Ltd., Tokyo, Japan); 2) cephalosporin antibiotics—cefazolin (CFZ; Astellas Pharma Inc., Tokyo, Japan), cefotiam (CTM; Nichi-Iko Pharmaceutical Co., Ltd., Toyama, Japan), ceftriaxone (CRO; Nichi-Iko Pharmaceutical Co., Ltd., Toyama, Japan), ceftazidime (CAZ; Tanabe Seiyaku Co., Ltd., Osaka, Japan), cefpirome (CPR; Myan Inc., PA, USA), cefepime (FEP; Bristol-Myers Squibb Co., NY, USA), and cefozopran (CZOP; Takeda Pharmaceutical Co., Ltd., Osaka, Japan); 3) carbapenem antibiotics; imipenem (IPM; Banyu Pharmaceutical Co., Ltd., Japan), and meropenem (MEM; Sumitomo Dainippon Pharma Co., Ltd., Osaka, Japan); and 4) cephamycin antibiotics and oxacephamycin antibiotics—cefmetazole (CMZ; Nichi-Iko Pharmaceutical Co., Ltd., Toyama, Japan) and flomoxef (FMOX; Shionogi & Co., Ltd., Osaka, Japan).

### Determination of the minimum inhibitory concentration (MIC)

Antimicrobial susceptibility tests for all bacterial strains were performed by the two-fold serial agar dilution method according to the Clinical Laboratory Standards Institute (CLSI) [17].

### Identification of β-lactamases

Class A (UOE-1, UOE-2, CTX-M-1, CTX-M-2, CTX-M-3, CTX-M-9, CTX-M-12, CTX-M-27, Toho-1, Toho-2, TEM, and SHV), Class B (IMP), Class C (CMY), DHA, and AmpR-DHA-1 β-lactamases were identified by Polymerase chain reaction (PCR) and DNA sequencing as reported previously [12].

### β-lactamase induction test

A 0.3-ml aliquot of each bacterial suspension (adjusted McFarland No. 0.5, approximately 1.5 × 10^8^ CFU/ml) was added to 10 ml of LB-broth and incubated with shaking at 35 °C for 1 h. Each β-lactam agent was added to the culture medium to make a 1/8 MIC final concentration, and then incubation was continued with shaking at 35 °C for 3–5 h. After rapid cooling, the cell pellets were harvested from each sample by centrifugation (4 °C, 15,000 rpm for 20 min), washed 3 times with 50 mM phosphate buffer (PB) (pH 7.0), resuspended with 2 ml in 50 mM PB, and then sonicated with an ultrasonic vibrator for 1 min in an ice bath. Centrifugation (4 °C, 15,000 rpm for 20 min) was performed again, and the supernatants were prepared as crude enzyme solutions for use in the β-lactamase assays.

### β-lactamase assays

The activities of β-lactamase were determined by spectrophotometer using nitrocefin (Nitrocefin; Kanto Chemical, Tokyo, Japan) [18]. A 10-μl aliquot of supernatant (crude enzyme solution) was added 990 μl of 100 μM nitrocefin solution, and the delta optical density (O.D.) of OD 486 nm was measured. The specific activities of β-lactamase were expressed in micromoles of nitrocefin hydrolyzed per minute per milligram of protein based on the changes in the absorbance at 486 nm at 35 °C for 3 min (ΔOD/sec). The supernatant protein concentrations were determined using the Bradford protein assay kit (TaKaRa Bradford Protein Assay Kit; TAKARA Bio Inc., Shiga, Japan). Bovine serum albumin (Bovine Serum Albumin; TAKARA Bio Inc., Shiga, Japan) was used as the standard. The ratio of the specific activities of β-lactamase and those of controls without antibiotics were used. The *E*. *coli* Mec5372 strain and *C*. *koseri* Rck4438 strain have co-production of TEM-1, and *K*. *pneumoniae* Rkp2004 strain have co-production of TEM-1 and SHV-12, so the β-lactamase activities of these strains were determined by a spectrophotometer using the nitrocefin solution after adding 4 μg/ml clavulanic acid (CVA; GlaxoSmithKline K.K., Tokyo, Japan).

## Results

### MICs

Tables 1 and 2 show the MICs for each strain. The MICs of PIP, PIP/TZB, CFZ, and CAZ ranged from 4–32 μg/ml, 2–16 μg/ml, 256 μg/ml, and 0.5–8 μg/ml in the reference group, respectively, and from 16–256 μg/ml, 4–64 μg/ml, 256–512 μg/ml, and 0.25–64 μg/ml in the DHA-1 group, respectively. The MICs were higher in the DHA-1 group than in the reference group. The MICs of fourth-generation cephems and carbapenems were relatively low (MIC ≤1 μg/ml), except for those of the *S*. *marcescens* ENsm22 strain, which had MICs for IPM of 4 μg/ml. The MICs for CTM, CRO, and FMOX in the reference and DHA-1 groups ranged from 16 to >128 μg/ml, 0.03–16 μg/ml, and 8 to >128 μg/ml, respectively, and from 4–32 μg/ml, 1–4 μg/ml, and 1–32 μg/ml, respectively. The MICs of these antibiotics in the DHA-1 group were lower than in the reference group.

**Table 1:** MICs for *Enterobacteriaceae* possessing chromosomally encoded *ampC*.

			MIC (μg/ml)												
	Plasmid	Status	PIP	PIP/TZB	CFZ	CTM	CRO	CAZ	CPR	FEP	CZOP	IPM	MEM	CMZ	FMOX
*E*. *cloacae*	ENel 67	Wild type	8	8	>128	>128	8	8	0.125	0.0625	0.125	0.25	0.0625	>128	64
*E*. *cloacae*	ENel 223	Wild type	8	4	>128	>128	4	2	0.125	0.0625	0.25	0.125	0.0625	>128	>128
*C*. *freundii*	Rcf 53	Wild type	4	8	>128	16	1	4	0.0625	0.0313	0.0625	0.5	0.0625	128	32
*S*. *marcescens*	ENsm 22	Wild type	16	8	>128	>128	4	2	2	1	2	4	0.25	>128	64
*S*. *marcescens*	ENsm 202	Wild type	32	16	>128	>128	16	1	2	0.5	1	0.5	0.5	>128	128
*M*. *morganii*	ENmm 80148	Wild type	4	2	>128	32	0.03	0.5	0.125	0.06	ND^a^	2	0.125	16	8

MIC, minimum inhibitory concentration; PIP, Piperacillin; PIP/TZB, Piperacillin/tazobactam; CFZ, Cefazolin; CTM, Cefotiam; CRO, Ceftriaxone; CAZ, Ceftazidime; CPR, Cefpirome; FEP, Cefepime; CZOP, Cefozopran; IPM, Imipenem; MEM, Meropenem; CMZ, Cefmetazole; FMOX, Flomoxef; *E*. *cloacae*, *Enterobacter cloacae*; *C*. *freundii*, *Citrobacter frundii*; *S*. *marcescens*, *Serratia marcescens*; *M*. *morganii*, *Morganella morganii*.

^a^: Not done

**Table 2:** MICs for *Enterobacteriaceae* producing inducible DHA-1 β-lactamase.

		MIC (μg/ml)												
	Plasmid	PIP	PIP/TZB	CFZ	CTM	CRO	CAZ	CPR	FEP	CZOP	IPM	MEM	CMZ	FMOX
*E*.*coli*	Mec 3968	16	4	>256	16	2	8	0.50	0.25	ND^a^	0.13	0.03	128	1
*E*.*coli*	Mec 5372	128	4	256	32	4	64	0.13	0.06	0.13	0.25	0.03	128	16
*K*. *pneumoniae*	Rkp 2004	256	64	>256	32	4	32	0.50	0.50	1	0.13	0.03	64	32
*C*. *koseri*	Rck 4438	16	4	256	4	1	0.25	0.25	0.13	0.13	0.06	0.02	256	4

MIC, minimum inhibitory concentration; PIP, Piperacillin; PIP/TZB, Piperacillin/tazobactam; CFZ, Cefazolin; CTM, Cefotiam; CRO, Ceftriaxone; CAZ, Ceftazidime; CPR, Cefpirome; FEP, Cefepime; CZOP, Cefozopran; IPM, Imipenem; MEM, Meropenem; CMZ, Cefmetazole; FMOX, Flomoxef; *E*.*coli*, *Escherichia coli*; *K*. *pneumoniae*, *Klebsiella pneumoniae*; *C*. *koseri*, *Citrobacter koseri*.

### Induction of β-lactamase by various antibiotics

Tables 3 and 4 show the induction of β-lactamase by several antimicrobials. The β-lactamase activities in the no-antibiotics group among controls were low (0.71–6.04 μmole of nitrocefin/min/mg) except for in the *E*. *coli* Mec5372 strain (15.31 μmole of nitrocefin/min/mg) and *E*. *cloacae* ENel67 strain (50.15 μmole of nitrocefin/min/mg). Regarding the relative β-lactamase activity, the induction of β-lactamase by PIPC, PIP/TZB, and third- and fourth-generations cephem was not observed except for with *K*. *pneumoniae* Rkp2004 by PIP/TZB (2.22) and *C*. *koseri* Rck4438 by FEP (2.74) in the DHA-1 group, and *C*. *freundii* Rcf53 by PIP (2.63) and *S*. *marcescens* ENsm22 by FEP (3.07) in the reference group. Furthermore, the induction ratios of chromosomal AmpC β-lactamase in the reference group by PIP/TZB were <1.51, and those of DHA-1 β-lactamase were <1.36, except for the *K*. *pneumoniae* Rkp2004 strain (2.22), which showed the highest MIC for PIP/TZB among all strains used in this study. β-lactamase induction by first- and second-generation cephalosporins, flomoxef, and carbapenems differed for all strains. CMZ strongly induced β-lactamase, except for with the *E*. *cloacae* ENel67 strain, which strongly produces AmpC without antibiotics (50.15).

**Table 3:** Induction of β-lactamase by various antibiotics in *Enterobacteriaceae* possessing chromosomally encoded *ampC*^a^.

			Relative β-lactamase activity													
	Plasmid	Status	No antibiotics	PIP	PIP/TZB	CFZ	CTM	CRO	CAZ	CPR	FEP	CZOP	IPM	MEM	CMZ	FMOX
*E*. *cloacae*	ENel 67	Wild type	1 (50.15)	0.88	1.20	1.45	1.94	1.51	1.16	1.45	1.30	1.64	1.20	1.36	1.32	1.37
*E*. *cloacae*	ENel 223	Wild type	1 (4.84)	0.93	0.58	3.87	5.45	1.19	1.31	1.14	0.82	0.72	12.01	16.67	26.66	15.78
*C*. *freundii*	Rcf 53	Wild type	1 (5.72)	2.63	1.51	1.72	1.83	1.29	2.43	1.81	2.14	1.41	4.58	6.13	8.62	5.48
*S*. *marcescens*	ENsm 22	Wild type	1 (6.04)	1.50	0.94	6.14	7.95	1.04	ND^b^	ND^b^	3.07	0.46	38.01	14.84	80.49	50.43
*S*. *marcescens*	ENsm 202	Wild type	1 (3.62)	1.22	1.28	2.02	2.98	1.07	1.29	1.13	1.04	0.75	6.21	2.56	3.57	4.08
*M*. *morganii*	ENmm 80148	Wild type	1 (0.74)	0.42	0.17	2.12	0.72	0.36	0.69	0.37	0.33	ND^b^	20.39	2.46	9.28	3.48

PIP, Piperacillin; PIP/TZB, Piperacillin/tazobactam; CFZ, Cefazolin; CTM, Cefotiam; CRO, Ceftriaxone; CAZ, Ceftazidime; CPR, Cefpirome; FEP, Cefepime; CZOP, Cefozopran; IPM, Imipenem; MEM, Meropenem; CMZ, Cefmetazole; FMOX, Flomoxef; *E*. *cloacae*, *Enterobacter cloacae; C*. *freundii*, *Citrobacter frundii; S*. *marcescens*, *Serratia marcescens; M*. *morganii*, *Morganella morganii*; Parenthesis: specific activities of various strains (μmole of nitrocefin/min/mg)

^a^: Each ß-lactam antibiotics was added into the culture medium to become 1/8 MIC of final concentration

^b^: Not done

**Table 4:** Induction of β-lactamase by various antibiotics in *Enterobacteriaceae* producing inducible DHA-1 β-lactamase^a^.

		Relative β-lactamase activity													
	Plasmid	No antibiotics	PIP	PIP/TZB	CFZ	CTM	CRO	CAZ	CPR	FEP	CZOP	IPM	MEM	CMZ	FMOX
*E*.*coli*	Mec 3968	1 (5.01)	1.04	0.47	0.96	1.19	1.33	1.02	1.41	1.13	ND^b^	3.03	1.46	10.29	1.56
*E*.*coli*	Mec 5372	1 (15.31)	0.99	0.98	1.20	1.39	0.84	0.98	1.05	0.94	0.97	1.58	1.32	3.26	1.98
*K*. *pneumoniae*	Rkp 2004	1 (1.25)	1.07	2.22	5.73	2.56	0.36	1.53	0.63	1.58	1.49	11.02	17.64	15.97	10.71
*C*. *koseri*	Rck 4438	1 (1.05)	1.08	1.36	5.30	1.40	1.18	0.98	1.42	2.74	ND^b^	2.56	1.43	35.63	2.33

PIP, Piperacillin; PIP/TZB, Piperacillin/tazobactam; CFZ, Cefazolin; CTM, Cefotiam; CRO, Ceftriaxone; CAZ, Ceftazidime; CPR, Cefpirome; FEP, Cefepime; CZOP, Cefozopran; IPM, Imipenem; MEM, Meropenem; CMZ, Cefmetazole; FMOX, Flomoxef; *E*.*coli*, *Escherichia coli*; *K*. *pneumoniae*, *Klebsiella pneumoniae*; *C*. *koseri*, *Citrobacter koseri*; Parenthesis: specific activities of various strains (μmole of nitrocefin/min/mg)

^a^: Each ß-lactam antibiotics was added into the culture medium to become 1/8 MIC of final concentration

^b^: Not done

## Discussion

There have been only few reports evaluating the induction of β-lactamase by several antibiotics using different bacterial strains under uniform conditions. Regarding the induction of β-lactamase in Gram-negative rods by β-lactam antibiotics, Sanders et al. [19] reported that cephamycin antibiotics and IPM had obvious induction of β-lactamase. In the present study which includes the DHA-1 group and the reference group, β-lactamase induction by first- and second-generation cephalosporins, flomoxef, and carbapenems differed for all strains. CMZ strongly induced β-lactamase, except for with the *E*. *cloacae* ENel67 strain. We also reported that the induction of β-lactamase induction by various antibiotics (including PIP/TZB) applied to various AmpC-producing *Enterobacteriaceae* strains (including DHA-1 producing strains) was low.

The AmpC β-lactamase of low-level producers, that is 0.74–6.04 μmole of nitrocfin/min/mg (no anitibiotics), were induced 2.56- to 38.01-fold and 3.57- to 80.49-fold by IPM and CMZ; however, it of high-level producers, that is 15.31 and 50.15 μmole of nitrocefin/min/mg (no anitibiotics), were induced 1.58- and 1.20-fold and 3.26- and 1.32-fold by IPM and CMZ. High-level producers might already have mutations in the *ampR* gene. Molecular biological research into the expression of AmpC and AmpR is needed in the future.

In the chromosomal AmpC reference group, the *E*. *cloacae* 67 strain, which is a higher producer, did not show any induction ability of β-lactamase, and the *E*. *cloacae* ENel 223 strain showed no induction ability by PIP or PIP/TZB but did show some β-lactamase induction by CFZ, CTM, CMZ, and carbapenems. Similar results were reported by Minami et al. [14]; those authors found that the *E*. *cloacae* GN5797 strain did not induce β-lactamase by PIP but did by CFZ, CTM, CMZ, CRO, and Latamoxef (LMOX). Another report by Goots et al. [20] stated that β-lactamase activity was highly induced by cephamycin C and LMOX in the *E*. *cloacae* 55 strain. In the report of Weber and Sanders [21], *Aeromonas Caviae* DLS4, *C*. *freundii* 21, *E*. *aerogenes* 76, *E*. *cloacae* 55, *M*. *morganii* 5, *P*. *aeruginosa* 164, and *S*. *marcescens* 1 did not induce β-lactamase by PIP at concentrations of 0.1, 1.0, 10, and 100 μg/ml. However, β-lactamase was induced by TZB (100 μg/ml) in *A*. *Caviae* DLS4, *C*. *freundii* 21, and *M*. *morganii* 5. β-lactamase was not induced by TZB at concentrations of 0.1, 1.0, or 10 μg/ml. TZB is β-lactamase inhibitor and high concentrations of TZB induce β-lactamase in some bacteria. These previous reports did not include PIP/TZB or fourth-generation cephems, and our data show the induction of β-lactamase by these antibiotics. Using *C*. *freundii* Rcf53, β-lactamase activity was induced by PIP, CAZ, FEP, carbapenems, CMZ, and FMOX, and the combined usage of PIP and TZB suppressed this induction. Akova et al. [22] showed that CVA induced β-lactamase, but TZB did not induce it using *C*. *freundii*. Weber and Sanders [21] showed that at 100 μg/ml, CVA, Sulbactam, and TZB induced β-lactamase, while concentrations of 0.1, 1.0, and 10 μg/ml did not. In addition, in our study, PIP did not induce β-lactamase in almost any bacterial species and strains, with only *C*. *freundii* Rcf53 showing induction. Further studies are necessary to confirm these results. AmpC β-lactamase in *S*. *marcescens* was induced by CFZ, CTM, FEP, carbapenems, CMZ, and FMOX. The degree of β-lactamase induction with the *S*. *marcescens* ENsm22 strain was higher than that with the *S*. *marcescens* ENsm202 strain. The *M*. *morganii* ENmm80148 strain showed β-lactamase induction by CFZ, CMZ, FMOX, and carbapenems. Previous reports [15,22] also showed that PIP and TZB did not induce β-lactamase using these bacterial species, but no data were available regarding cephamycin, oxacephem and carbapenem antibiotics [23].

Thus far, the published data on DHA-1 β-lactamase induction by various antibiotics in many clinical strains of *Enterobacteriaceae* are insufficient. The β-lactamase induction of the DHA-1 group by PIP, PIP/TZB, and third- and fourth-generation cephems were not observed, except for with the *K*. *pneumoniae* Rkp2004 strain by PIP/TZB (2.22-fold) and the *C*. *koseri* Rck4438 strain by FEP (2.74-fold). The trends in β-lactamase inductions of the DHA-1 group were similar to those in the chromosome-mediated reference group. The β-lactamase in the DHA-1 group originated from chromosomal AmpC in *M*. *morganii* [9], but the induction of β-lactamase differed among bacterial species and strains. In particular, regarding the induction of β-lactamase by CMZ, β-lactamase was strongly induced in the *C*. *koseri* Rck4438 strain, but β-lactamase was only weakly induced in the *E*. *coli* Mec5372 strain.

β-lactamase induction by PIP and PIP/TZB was not observed in either the DHA-1 or reference groups, except for *K*. *pneumoniae* Rkp2004 by PIP/TZB and *C*. *freundii* Rcf53 by PIP. β-lactamase induction by PIP/TZB tended to be weaker than that induced by PIP. Kadima et al. [24] reported that TZB was not associated with the inhibition of β-lactamase induction and only worked to remove chromosomal AmpC β-lactamase already induced by β-lactam antibiotics. For similar reasons, the β-lactamase activity induced by PIP/TZB was lower than that induced by PIP alone in the present study.

The outbreak by DHA-1 producers has been reported [12], and DHA-1 has been detected in relatively healthy outpatients [13]. In addition, part of the IncL/M plasmids were DHA-1-encoding plasmids in *K*. *pneumoniae* and the presence of DHA-1-encoding plasmids could allow for the wide distribution among other species of *Enterobacteriaceae* as IncL/M OXA-48 plasmids are observed not only in *K*. *pneumoniae* but also in *E*. *coli* [25]. Thus, it is important to look for plasmid genes that include DHA-1 in order to prevent them from spreading.

On the other hand, this study has limitations. The β-lactamase assays were not performed more than once on each sample due to laborious process. But, these data are valuable because it is important to compare the difference of the DHA-1 β-lactamase-based induction by each antibiotic. So, these results would become important information when proper antibiotics are selected in infection. MICs with PIP/TZB were twice lower than those with PIP in the reference group (Table 1) while those with PIP/TZB were about 4 times lower than those with PIP in the DHA-1 group (Table 2). We do not know why the MIC with PIP/TZB was higher in the reference group than in the DHA-1 group. It is possible that TZB inhibited the resistance mechanism in the DHA-1 groups that did not receive β-lactamase inhibitors.

## Conclusion

In the present study, we measured the activity of β-lactamase with and without several types of β-lactams using several types of bacterial species and strains under uniform conditions. In addition, this study demonstrated that the induction of DHA-1 β-lactamase was similar to that of chromosomal AmpC β-lactamase using various Enterobacteriaceae. Most DHA-1 β-lactamase is encoded via plasmid. Therefore, various *Enterobacteriaceae* species are able to acquire DHA-1, and the outbreak by DHA-1-producing *Enterobacteriaceae* might occur in clinical settings. We must ensure we understand the induction of DHA-1 β-lactamase of various antimicrobial agents.
